# Pattern of care and treatment outcome of high-grade glioma patients at 4 Ethiopian teaching hospitals: a prospective cohort study

**DOI:** 10.1093/noajnl/vdag207

**Published:** 2026-08-10

**Authors:** Dawit Workneh Gechu, Tsegazeab Laeke Tekelemariam, Bethelhem Yesehak Worku, Mehari Wale Alem, Yordanose Girma Legesse

**Affiliations:** Neurosurgery Unit, Department of Surgery, University of Gondar, Gondar, Ethiopia; Neurosurgery Unit, Department of Surgery, Addis Ababa University, Addis Ababa, Ethiopia; Neurosurgery Unit, Department of Surgery, Addis Ababa University, Addis Ababa, Ethiopia; Department of Neurosurgery, Saint Paul’s Hospital Millennium Medical College, Addis Ababa, Ethiopia; Aletha Medical Center, Addis Ababa, Ethiopia

**Keywords:** brain tumor, GBM, high grade glioma, LMIC

## Abstract

**Background:**

High-grade gliomas are the most prevalent type of malignant brain tumor in adults and the second leading cause of cancer-related death. This study assesses epidemiology, pattern of care, and treatment outcome of high-grade glioma in Ethiopia.

**Methods:**

A Prospective cohort study. Data collected were analyzed using SPSS. Frequency distribution was used to describe the characteristics of the study participants. Chi-squared tests and survival analysis were used to assess the treatment outcomes. Variables with *P* < .05 were considered as significant.

**Results:**

There was a total of 40 patients. The median age at diagnosis was 47 years. Headache was the commonest presenting symptom (97.5%), followed by seizure (52.5%) and weakness (50%). The pre-operative tumor volume ranged between 3.9 and 168 cm^3^, with a mean volume of 80.38 cm^3^. A total of 22 patients had ≥97% extent of resection. About 17.5% of the patients died post-treatment. The mean survival time was 20.77 months (95% CI: 18.46-23.09 months). There was significant association with higher Karnofsky performance status (KPS) scale (≥70), pre-operative GCS, and post-operative adjuvant therapy with survival outcome (χ^2^ = 10.19 *P* = .006, χ^2^ = 12.9 *P* = .005, χ^2^ = 6.716 *P* = .035), respectively.

**Conclusion:**

In our study, the mean survival time was 20.77 months. Higher KPS, pre-operative Glasgow Coma Scale (GCS) of 15, and patients receiving post-op adjuvant therapy had a significant favorable association with survival in our study.

Key Points

- Higher KPS, pre-operative GCS of 15, and receiving post-op adjuvant therapy had a significant favorable association with survival.
- The mean survival time was 20.77 months.
- About 57.5% of the patients had good functional recovery with Glasgow outcome scale (GOS 5) at 2 years follow up.

Importance of the Study

This research proposal represents the first study of its kind in Ethiopia, aiming to address critical knowledge gaps in the existing treatment patterns of high-grade glioma (HGG) patients and the management of associated complications. This study will generate pioneering data to inform evidence-based strategies optimizing HGG management within the Ethiopian context and contribute to improved survival rates and quality of life for HGG patients.

High-grade gliomas (HGG), particularly glioblastoma multiforme (GBM), represent the most aggressive primary brain tumor, and its incidence continues to rise globally.^1^^,^^2^ While advancements in multimodal treatment, including surgery, radiotherapy, and chemotherapy, have improved prognoses in high-resource settings, disparities in outcomes persist, particularly in low- and middle-income countries (LMICs) such as Ethiopia.

## Rationale of the Study

This research proposal aims to address critical knowledge gaps in the existing treatment patterns of HGG patients and management of associated complications by comprehensively investigating the patterns of care and treatment outcomes for HGG patients at a tertiary teaching hospital and its affiliate Hospitals in Ethiopia. The findings will provide valuable data to inform evidence-based strategies for optimizing HGG management within the Ethiopian context, ultimately contributing to improved survival rates and quality of life for HGG patients in this underserved region.

## Objectives

### General Objectives

To assess the pattern of care, treatment outcomes, and associated factors of high-grade glioma patients found at Tikur Anbessa Specialized Hospital and affiliated neurosurgical hospitals in Addis Ababa, Ethiopia, from September 1, 2023 to November 1, 2025.

### Specific Objectives

To assess the pattern of care for patients, treatment outcomes found, and determine predictors of treatment outcome at Tikur Anbessa Specialized Hospital and affiliated neurosurgical hospitals diagnosed with high-grade glioma in Addis Ababa, Ethiopia, from September 1, 2023 to November 1, 2025.

## Methods

### Study Design

Prospective cohort Study.

### Study Setting

Tikur Anbessa Hospital or Black Lion Hospital in Addis Ababa, Ethiopia, was established in 1964. It serves as the main teaching hospital for both preclinical and clinical training across various disciplines in the School of Medicine at Addis Ababa University.

### Study Participants

All patients who had been diagnosed with MRI and pathology as WHO grade 3 and 4 high-grade glioma patients at the selected hospitals who fulfilled the inclusion criteria.

### Histopathology Classification

Tumor classification was based on histopathological examination of surgical specimens performed at the pathology departments of the participating institutions. Specimens were evaluated using routine hematoxylin and eosin staining, and tumors were graded based on established histological criteria consistent with high‑grade gliomas (WHO Grade III and IV), including cellular atypia, mitotic activity, microvascular proliferation, and necrosis.

Molecular analyses required for the integrated diagnosis according to the 2021 World Health Organization (WHO) classification of central nervous system tumors—such as IDH mutation status and other molecular markers—were not routinely available during the study period due to resource limitations.^3^^,^^4^ Therefore, tumors were classified based on histology alone, and all cases were collectively reported as high‑grade gliomas (HGGs). The specific histologic subtypes identified will be classified using the “Not Otherwise Specified” (NOS) designation (eg, Astrocytoma, NOS), as outlined by the 2021 WHO Classification.^3^ This approach reflects current clinical practice in many resource‑limited settings and was adopted to ensure internal consistency across participating centers.

### Inclusion Criteria

All patients who were newly confirmed high-grade glioma patients (WHO grade 3 and 4) diagnosed with MRI and pathology, under treatment for this diagnosis during the study period, will be included in this study.

### Exclusion Criteria

Those patients with incomplete medical records were excluded from the study.

All patients who were unwilling to participate and lost to follow-up in this study will be excluded.

### Exposure

The variables assessed were age, KPS, histopathology, WHO grade, extent of resection (EOR), extent of residual tumor, post-op adjuvant treatment, and functional outcome.

### Outcome

The primary outcome was overall survival and progression-free survival defined at 2 years following treatment.

### Statistical Analysis

Data were entered after it was checked for completeness into SPSS and analyzed. Descriptive analysis was done using frequency, mean, median, and standard deviation for all the important variables. Chi-square test was done to assess association between categorical variables. Variables with *P* < .05 were considered significant. Survival and ­treatment success probabilities were estimated using Kaplan-Meir method.

### Ethical Consideration

Before data collection to conduct this study, ethical clearance was obtained from the institutional review board (IRB) at Black Lion Specialized Hospital and submitted to MCM (Myung Sung Christian Medical Center), Zewditu Memorial Hospital, and Alert Specialized Hospital research affairs. The aim of the study was clearly explained to the study participants, and their right to refuse was maintained. Information was collected after obtaining informed verbal consent from each participant’s caregivers. The personal information of study participants was kept entirely anonymous, and confidentiality was assured throughout the study period. The name and address of the patient were omitted from the questionnaire.

## Results

### Socio-Demographic Characteristics

There were 40 patients with confirmed HGG who participated in this study. The minimum follow-up time was 23 days, and the maximum amount of time patients were followed in this study was 362 days. Among the study participants, the minimum age was 10 years old and the maximum age was 75 years old, with the median age at diagnosis being 47 years. Most of the participants (35%) in this study were 50 years old and above. The male-to-female ratio was 1.85:1. More than half (*n* = 21, 52.5%) of the patients who participated in this study were from Addis Ababa; the remaining participants were from other regions of the country (Table 1). Fifty percent (*n* = 20) of study participants were operated at MCM Hospital and 40% (*n* = 16) at BLH. The majority of the patients (*n* = 37, 92%) were right-handed.

**Table 1.** Socio-demographic characteristics of patients

Socio-demographic characters	Frequency (*N* = 40)	Percent
Age: <30 years	5	12.5%
30-40 years	8	20%
41-50 years	13	32.5%
≥50 years	14	35%
Sex: Male	26	65%
Female	14	35%
Hospital operated		
Black Lion	16	40%
MCM	20	50%
Zewditu	3	7.5%
Alert	1	2.5%

### Pre-Operative Presentation

The median duration of symptoms prior to presentation to hospital was 3 months (IQR 2-7.5 months). There was no significant association between WHO grade and duration of symptom prior to presentation (*χ*^2^ = 6.77, *P* = .486).

The majority of the patients (*n* = 33, 82.5%) had pre-operative GCS of 15. Headache is the commonest presenting symptom in this study (*n* = 39, 97.5%), followed by seizure (*n* = 21, 52.5%) and weakness (*n* = 20, 50%) (Table 2).

**Table 2.** Frequency distribution of pre-operative presentation of patients

	Frequency (*N* = 40)		Percent
Symptoms			
Headache	39		97.5%
Seizure	21		52.5%
Weakness	20		50%
Behavioral change	9		22.5%
Decreased mentation	3		7.5%
Vomiting	2		5%
Speech difficulty	7		17.5%
Visual complaint	4		10%
Cranial nerve deficit	1		2.5%
Pre-operative GCS			
9	1		2.5%
10	1		2.5%
14	5		12.5%
15	33		82.5%
KPS			
10-60	4		10%
70-80	28		70%
≥90	8		20%
Tumor location			
Frontal	19		47.5%
Temporal	11		27.5%
Parietal	8		20%
Midline—corpus callosum	2		5%

The commonest comorbidity identified was hypertension (*n* = 2, 5%), followed by Diabetes (*n* = 1, 2.5%). Only one patient had ionizing radiation treatment for breast cancer following surgery, identified as a risk factor for developing HGG.

Most of the cases (*n* = 28, 70%) presented with KPS of 70-80% followed by KPS ≥90 in 20% (*n* = 8) of the patients.

### Tumor Location

Most of the patients (*n* = 19, 47.5%) in this study had the tumor located in the frontal area, followed by the temporal area. There was no significant association between WHO grade and tumor location in this study (*χ* ^2^ = 1.847, *P* = .7). There is an equal distribution of tumors across the two hemispheres. Only 2(5%) of the patients had isolated midline (corpus callosum) HGG.

### Pre-Operative Tumor Volume and Number of Sites Involved

The pre-operative tumor volume among the study participants ranged between 3.9 and 168 cm^3^. The mean pre-operative tumor volume was 80.38 cm^3^ ± SD 42.9 cm^3^. Most of the patients (*n* = 35, 87.5%) in this study had a single-focus lesion, whereas the rest (*n* = 5, 12.5%) had multiple foci.

### Pre-Operative Medications

All patients were given corticosteroids before surgery. Among the 40 patients who participated in the study, 45% (*n* = 18) had received prophylactic anti-seizure medications. The remaining 55% (*n* = 22) of the patients who had seizures preoperatively had received therapeutic anti-seizure medication. Unfractionated heparin or other prophylactic anticoagulants were given for 35% (*n* = 14) of patients.

### Type of Intervention

Among the patients, 90% (*n* = 36) of them had primary surgery, whereas 10% (*n* = 4) had re-operation for recurrent WHO grade 4 Astrocytoma NOS. The mean time to intervention after initial diagnosis was 16 weeks.

### Extent of Resection

A total of 22.5% (*n* = 9) of the patients had extent of resection between 70 and 96% and 55% (*n* = 22) of the patients had ≥97% extent of resection. About 20% (*n* = 8) of the cases had biopsy. Intraoperative ultrasound was used to maximize EOR in only 17.5% (*n* = 7) of the patients. Surgeon’s estimate of extent of tumor resection is significantly associated with the objective EOR (*χ*^2^ = 32.759, *P* = .0000).

### Post-Operative Residual Tumor Volume and Complications

After surgery, 23 of the patients (57.5%) had post-op residual tumor volume of <5 cm^3^ with median tumor volume of 1.96 cm^3^ (IQR 0-21 cm^3^). The commonest (*n* = 11, 27.5%) post-operative complication was worsening and/or new neurological deficit. About 5% (*n* = 2) of the patients developed thromboembolic (PE and/or DVT) complications. One patient (2.5%) had tumor bed hematoma (wounded glioma).

### Histopathology

The commonest tumor histology (85%) seen in this study is astrocytoma NOS, followed by oligodendroglioma NOS (12.5%). Only one patient had pleomorphic xanthoastrocytoma NOS. 72.5% of the patients had a tumor with WHO grade 4 and the remaining 27.5% were WHO grade 3. Only 1 (2.5%) of the study participants had a molecular study with WHO grade 3 Astrocytoma IDH mutant.

### Post-Operative Adjuvant Therapy

A total of 57.5% (*n* = 23) of the patients received post-operative adjuvant treatment as radiation (RT) with (*n* = 8, 20%) or without (*n* = 17, 42.5%) temozolamide as per Stupp protocol. The remaining 42.5% (*n* = 17) of patients didn’t receive any adjuvant treatment.

### Survival

**Figure 1.**
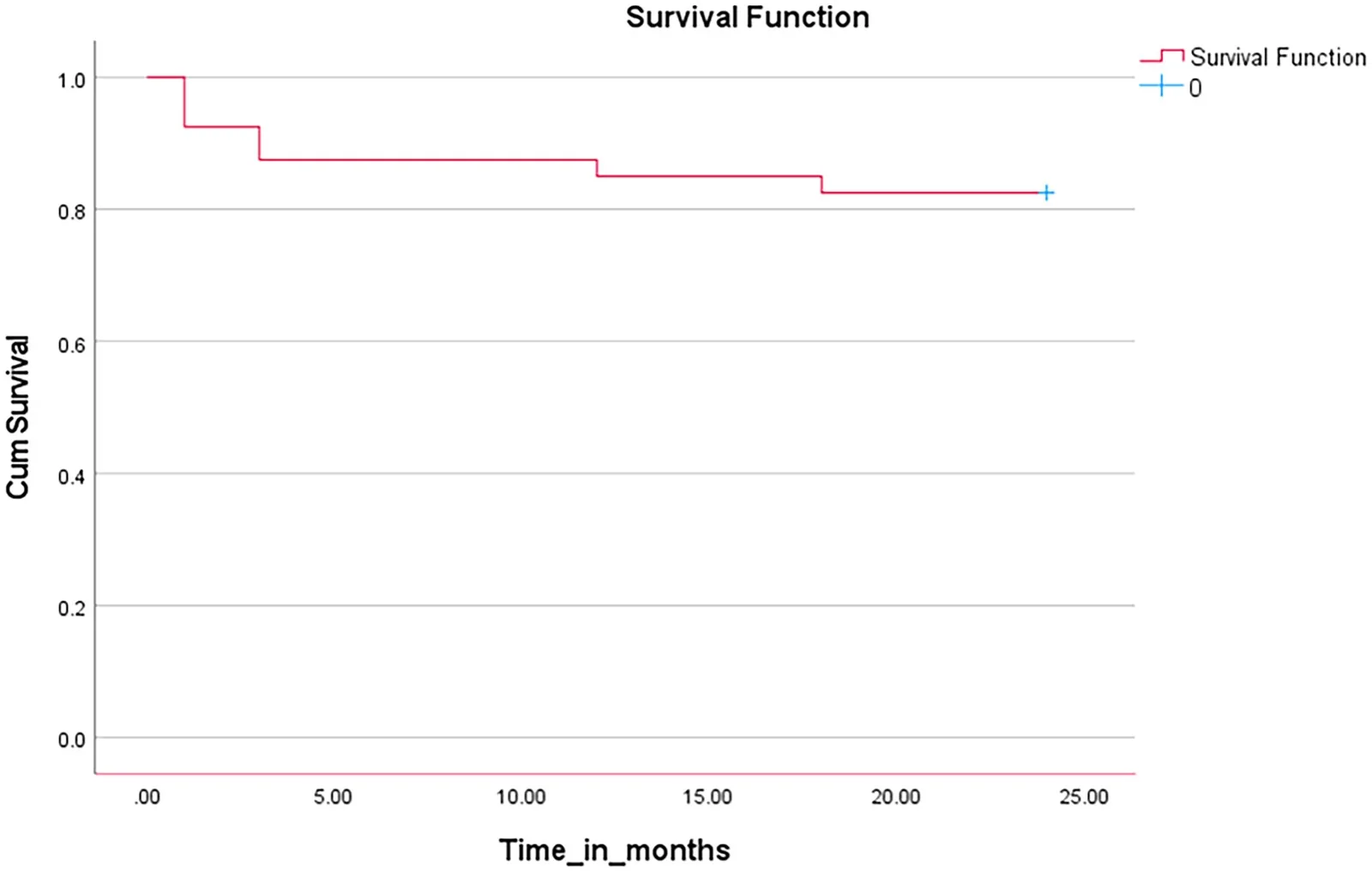
Kaplan-Meier survival curves for overall Survival of patients with HGG post-OP.

In this study, 17.5% (*n* = 7) of the patients diagnosed with HGG died post-surgery, of whom 71.4% had a WHO grade 4 tumor. The mean overall survival time for patients with HGG post-surgery was 20.7 months (95% CI: 18.46-23.09 months) (Figure 1).

**Figure 2.**
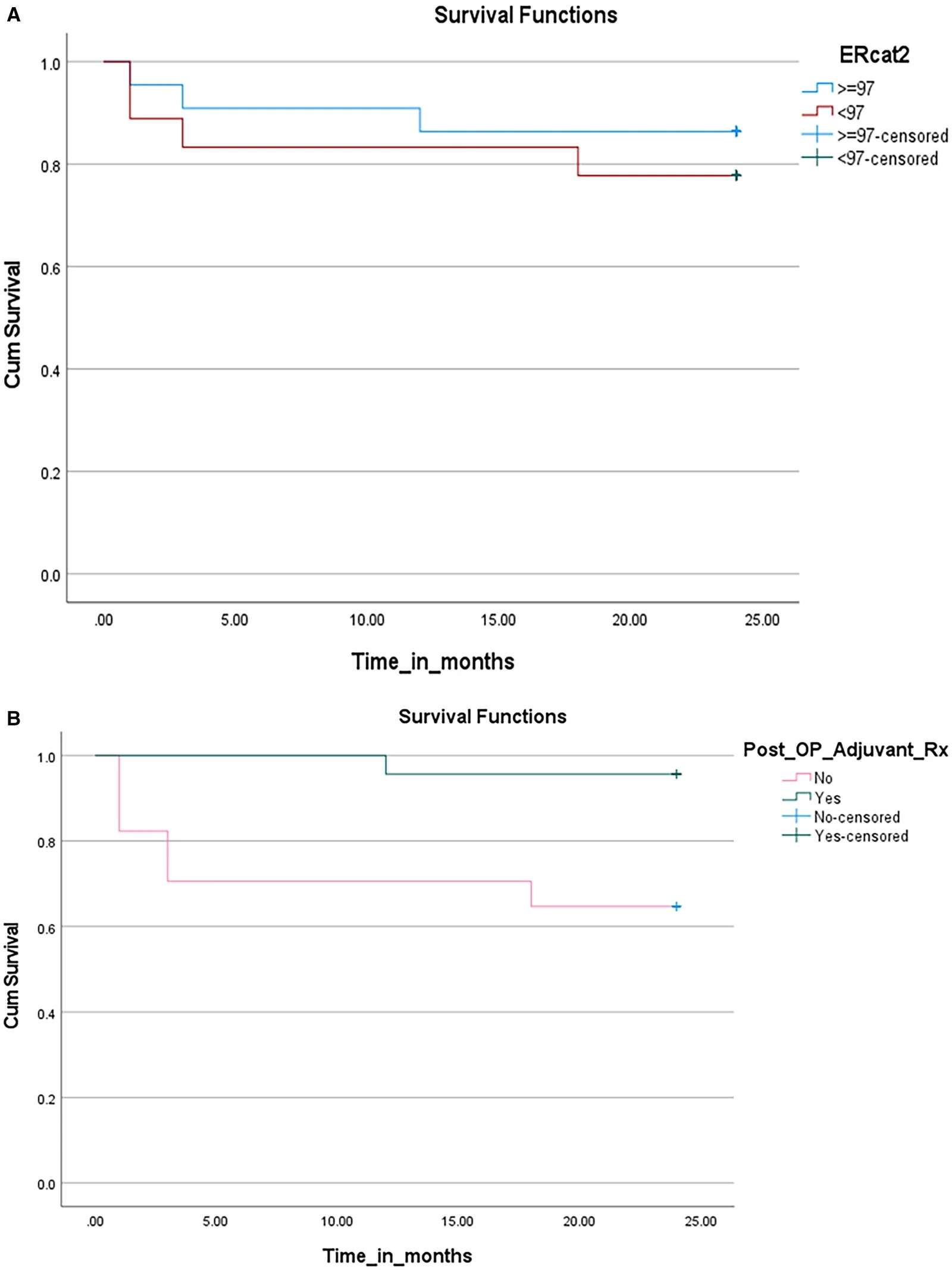
(A) Kaplan-Meier survival curves stratified by extent of resection figure. (B) Kaplan-Meier survival curve analysis by receipt of post­operative adjuvant therapy.

There was a significant association between patients with higher KPS (≥70) value and their survival outcome. (*χ*^2^ = 10.19 *P* = .006). Patients with pre-operative GCS (≤14) seem to have a significant association with survival outcome compared with patients having GCS of 15. (*χ*^2^ = 12.9 *P* = .005). Those patients who received post-operative adjuvant therapy had better survival outcomes than those patients with no adjuvant therapy. (*χ*^2^ = 6.716) *P* = .035) (Figure 2B). There was no association between survival and WHO grade (Table 3).

**Table 3.** Chi-square test for KPS, pre-operative GCS, and post-adjuvant therapy compared with survival

	Survival		χ^2^	*P*-value
	Alive (*n* = 33)	Dead (*n* = 7)		
Age				
<30 years	3	2	3.458	.284
30-40 years	8	0		
41-50 years	11	2		
≥50 years	11	3		
KPS				
10-60	1	3	10.19	**.006**
70-80	25	3		
**≥90**	7	1		
GCS				
9	0	1	12.79	**.005**
10	0	1		
14	3	2		
15	30	3		
WHO grade				
3	9	2	0.005	.944
4	24	5		
Post-op adjuvant				
RT	12	1	6.768	**.009**
RT + TMZ	10	0		
No RT + TMZ or RT	11	6		

Bold values: *P* < 0.05.

The mean survival time for those patients with extent of resection ≥97% is 21.45 months (CI: 18.67-24.23 months, *P* = .001) (Figure 2A). The mean survival of patients who received adjuvant treatment is 23.47 months with log rank off (*P* = .01, CI: 22.47-24.47 months). More than half (57.5%) of patients had a good functional outcome (GOS 5) at 2-year follow-up (Table 4).

**Table 4.** Functional status at 12 and 24 month post OP

GOS	12 month		24 month	
	Frequency	Percent	Frequency	Percent
1—Death	6	15%	7	17.5%
2—Persistent vegetative state				
3—Severe disability	4	10%	5	12.5%
4—Moderate disability	7	17%	5	12,5%
5—Good recovery	23	57%	23	57.5%
Total	40	100%	40	100%

There was no significant association seen between functional status (GOS) of patients and extent of resection in this study. Similarly, no association was found between functional status (GOS) and having post-adjuvant treatment.

## Discussion

In this study, the findings showed clinical characteristics of operated HGG patients preoperatively and postoperatively. This research also estimated the survival outcome of these patients after treatment.

The treatment outcomes for 40 patients diagnosed with high-grade gliomas (HGG) were evaluated over a two-year period, commencing September 1, 2023, and concluding September 1, 2025. The mean survival duration following surgery was recorded at 20.77 months, with 7 patients (17.5%) having succumbed by the end of the observation period. Notably, the mean length of survival in this analysis was higher compared to findings in other studies.^5-7^

This investigation indicated that the most affected age group for HGG was individuals aged 50 years and above, a finding consistent with research conducted in the U.S. ­concerning prognostic indicators of survival in glioblastoma patients. Various studies suggest that a younger age correlates significantly with enhanced survival outcomes.^1^^,^^8-10^ However, this study did not find a significant link between age and survival rates. A higher male-to-female ratio was observed, aligning with results from ­previous research.^6^^,^^11^

The predominant initial symptoms reported were headaches and seizures, corroborating findings from a U.S. study on care patterns for adults newly diagnosed with malignant glioma.^12^ This research revealed that solitary lesions were more prevalent than multifocal ones, representing 87.5% of cases. This observation aligns with that of Dr. Akinmoladun and colleagues, who examined MRI patterns of glioblastoma in a tertiary care setting.^13^

A majority of the patients (70%) had a Karnofsky Performance Status (KPS) score between 70 and 80, while 20% achieved scores of 90 or higher, and 10% fell between scores of 10 and 60. The Glasgow Coma Scale (GCS) recorded a score of 15 in most cases (82.5%). These results are consistent with a Nigerian study investigating the challenges related to the management of high-grade gliomas. A notable difference in patient outcomes was found between those with KPS scores lower than 60 compared to those with scores greater than 90, a finding that aligns with previous research conducted in Nigeria.^2^

In contrast to other studies, this investigation did not establish a significant relationship between the World Health Organization (WHO) grade and survival outcomes.^7^^,^ ^14^ The absence of a significant association between survival and WHO grade in this study contrasts with reports from high‑income settings. This finding may be attributed to the lack of molecular classification, potential histological overlap between grades, and the dominant influence of treatment access and extent of resection on survival in resource‑limited settings. Additionally, the small number of WHO Grade III cases may have limited the ability to detect statistically significant survival differences. These findings suggest that, in LMIC contexts, health system and treatment factors may play a more critical role in patient outcomes than histological grade alone.

The frontal lobe (47.5%) was identified as the most common tumor location, followed by the temporal (27.5%) and parietal (20%) lobes. These results are in agreement with a Bulgarian study that focused on the clinical aspects and prognostic factors of HGG surgery.^11^

The mean preoperative tumor volume was calculated at 80.38 cm³ (range 3.9-168 cm³), with 52.5% of cases achieving an extent of resection (EOR) of 97% or higher. This is comparable to a U.S. study examining the prognosis, extent of resection, and survival in glioblastoma patients, albeit with a higher mean preoperative tumor volume.^15^ EOR of ≥97% is frequently associated with improved survival ­outcomes in numerous studies; similarly this study has shown a statistically significant survival difference between patients with EOR ≥97% and those with EOR <97% (*P* = .01).^11^^,^^15-17^

Postoperative adjuvant therapy, either radiation (RT) or temozolomide (TMZ), was administered to 57.5% of the patients. This figure is notably lower than that reported by Joanna et al,^7^ who found that 68% of patients received postoperative adjuvant therapy following radiation treatment for HGG. The reduced percentage in the present study may be attributed to the expense associated with TMZ and the lengthy waiting periods for radiation therapy. A significant difference was noted in survival outcomes between patients who received adjuvant therapy following surgery and those who did not.^5^^,^^18^ Similar results were observed in a Nigerian study addressing the challenges in managing high-grade gliomas.^2^

A clinical review by Antonio on glioblastoma and other malignant gliomas indicated that venous thromboembolic events occur in 20-30% of patients, contrasting with this study, where only 5% of patients experienced such events.^19^

### Strength

This research assessed the treatment outcome and pattern of care of HGG patients during the study period in a detailed manner. I believe this research provides valuable information on patients’ characteristics and outcomes after surgery.

### Limitation

The lack of molecular profiling represents an important limitation of this study. While integrated WHO 2021 classification could not be applied, our histology‑based approach reflects real‑world practice in many LMICs.

Due to resource limitations, tumor volumes were estimated using the simplified ellipsoid formula (V = A × B × C/2), which assumes a uniform shape and may overestimate the true volume of irregularly shaped gliomas compared to 3D volumetric segmentation.

Limited access to radiotherapy facilities, chemotherapy availability, financial constraints, and treatment delays significantly affected the delivery of guideline‑recommended care, treatment outcomes and limit direct comparison with reports from high‑income settings.

The psychosocial and detailed functional (quality of life) outcomes of the patients was also not assessed.

### Recommendations

This study highlights the substantial challenges in managing high‑grade glioma in resource‑limited settings, where outcomes appear to be driven more by access to surgery and adjuvant therapy than by histological grade alone. Our findings emphasize the need for earlier referral, prioritization of maximal safe resection through targeted neurosurgical capacity building, and improved access to radiotherapy and chemotherapy to enhance treatment completion. Coordinated action by policymakers, healthcare institutions, and international partners is urgently needed to strengthen neuro‑oncology infrastructure, expand multidisciplinary care, and incrementally build molecular diagnostic capacity in LMICs to improve patient outcomes.

## Conclusion

High-grade glioma (HGG) represents the most aggressive primary brain tumor. In this study, we had 40 patients who were diagnosed with HGG with histopathologic confirmation. The mean overall survival time for patients with HGG post-surgery was 20.7 months (95% CI: 18.46-23.09 months). There was a significant association between survival outcome and preoperative KPS, preoperative GCS, and receiving postoperative adjuvant therapy.

## Data Availability

The data that support the findings of this study are available from the corresponding author upon reasonable request. Due to ethical restrictions, the data are not publicly available. However, de-identified datasets and supporting materials can be shared for academic research purposes upon approval. Requests for access should be directed to the corresponding author’s email.
